# Maternal Parenting Styles and Glycemic Control in Children with Type 1 Diabetes

**DOI:** 10.3390/ijerph16020214

**Published:** 2019-01-14

**Authors:** Riitta Hannonen, Kaisa Aunola, Kenneth Eklund, Timo Ahonen

**Affiliations:** 1 Kymsote—Kymenlaakso Social and Health Services, Department of Psychology, Kotkantie 41, FI-48210 Kotka, Finland; 2 Department of Psychology, University of Jyväskylä, PL 35, FI-40014 Jyväskylä, Finland; kaisa.aunola@jyu.fi (K.A.); kenneth.m.eklund@jyu.fi (K.E.); timo.p.s.ahonen@jyu.fi (T.A.)

**Keywords:** children, type 1 diabetes, glycemic control, mothers, parenting style

## Abstract

The purpose of this study is to examine differences in parenting styles between mothers of children with type 1 diabetes and mothers of healthy children and to explore relationships between parenting styles and glycemic control of children with diabetes. Mothers of 63 children with diabetes and mothers of 83 children without diabetes reported their parenting styles using the Blocks’ Child Rearing Practices Report, when their child was 9–10 years old. Glycemic control of the children with diabetes was evaluated 1 year after diagnosis (<6 years of age) and at the time of the study (at 9–10 years). Mothers of children with diabetes used more psychological control than mothers of healthy children. Among girls with diabetes, poorer early glycemic control was associated with mothers’ subsequent greater use of psychological control. Behavioral control was positively associated with poorer current glycemic control. In boys, psychological control was positively associated with poorer current glycemic control. Psychological control in families with diabetes needs attention, because it has shown to be associated with poorer diabetes care.

## 1. Introduction

Type 1 diabetes (T1D) care is based on frequent monitoring of blood glucose and administration of insulin adjusted to blood glucose level, meals, exercise, and health. In a young child with T1D, parents bear the responsibility of the treatment, which requires continuous monitoring of the child. Since the complications of diabetes are life threatening, parents often experience distress and anxiety over their child’s well-being [1]. Because parents are deeply involved with their child’s care, they may be in danger of becoming overprotective and controlling of their child [2]. Management of diabetes self-care behaviors transfers gradually from being parent-directed to shared responsibility of the parents and the school-age child and should be fitted to the child’s developmental level. Sometimes parenting and interaction between the child and the parents are affected by these new demands of the child’s autonomy. The present study examines parenting styles in mothers of school-age children with T1D and their relationship with the children’s diabetes care.

Parenting styles refer to the stable attitudes, values and behaviors of a parent towards her child [3]. In the literature, three parenting style dimensions have been used to describe individual differences in parenting behaviors: parental warmth, behavioral control and psychological control [4,5]. Parental warmth and affection refers to the extent to which parents show affective warmth, acceptance and involvement in their interaction with their child [5]; behavioral control refers to parenting behaviors that are intended to control the child’s behavior (e.g., limit setting, maturity demands); whereas psychological control is exercised over thoughts and emotions of the child using psychological means, such as guilt induction, manipulation and love withdrawal [6,7].

High levels of parental affection and behavioral control, as well as their combination in terms of an authoritative parenting style, have been shown to have a positive effect on a child’s adjustment [5], whereas a high level of psychological control has been associated with lower self-esteem, internalizing problems and behavioral problems [6,8,9,10]. These results concerning the associations of parenting styles with child well-being have been found to be true also among children with T1D: A supportive and warm parenting style has been found to be associated with better quality of life [11,12] and fewer symptoms of depression [13,14] in adolescents with T1D, whereas psychological control has been shown to be associated with depressive symptoms [13] and externalizing behavior [15]. It has also been found that adolescents with T1D are more sensitive to psychological and behavioral control than younger children with T1D [13], which may result in poorer quality of life, especially among girls [16].

Some studies link parenting styles with diabetes care, although associations between parenting styles and glycemic control have not always been found [15,17,18,19]. For example, better adherence in diabetes care has been associated with an authoritative parenting style characterized by both warmth and behavioral control in children [18,20] and in adolescents [12,21,22]. Moreover, parental monitoring and involvement, which resembles behavioral control, has been found to relate to better adherence and glycemic control in adolescence [14,16,23], but critical parenting (a concept similar to psychological control) has been associated with poorer self-efficacy and self-care [24]. In older adolescents, parental control (psychological and behavioral control not differentiated) has been associated with poorer adherence [16], highlighting the need for parents to adjust their level of control to the developmental stage of the child [25].

Although there are some studies focusing on the role of parenting styles in children’s well-being and diabetes care among children with T1D, this earlier research has some limitations. First, most previous studies have used only clinical samples without a comparison group. Therefore, although it has been reported that most parents of children with T1D use parenting characterized by both a high level of warmth and behavioral control (i.e., authoritative parenting style [18,19]), it is not known whether their parenting behaviors differ from those of parents with children without diabetes. To our knowledge, only one study compares parenting styles between parents with a child with T1D and parents with healthy children. In this earlier study, adolescents with T1D reported their parents to control them more than parents of healthy children [17]. Because parenting styles in families with younger children with and without T1D have not been compared before, it is not known whether the results found by Graue and colleagues [17] are specific for the period of adolescence only. Although psychological control seems to be relatively common in families with a chronic disease [26], the relationship between parents’ use of psychological control and diabetes care is not yet well understood.

The aims of the present study were twofold. The first aim was to compare parenting styles of mothers of school-age children with early-onset T1D and mothers of healthy children. The hypothesis was that the mothers of children with T1D use more psychological and behavioral control than the mothers of healthy children [17,26]. The second aim was to examine the relationships between the mother’s parenting styles and the child’s glycemic control at different time points during childhood in children with T1D. The hypothesis was, first, that a child’s poor glycemic control in early childhood is associated with the mother’s subsequent high use of psychological control. Second, maternal psychological control was hypothesized to be related to child’s poorer concurrent glycemic control, while maternal behavioral control and affection were hypothesized to be related to having better concurrent glycemic control.

## 2. Materials and Methods

### 2.1. Sample and Design

The children took part in a large cross-sectional study assessing academic skills and cognitive and psychosocial functioning in children with early-onset T1D [27]. All the children (*n* = 79) who fulfilled the inclusion criteria (type 1 diabetes diagnosed before 5 years of age and current age between 9–10 years at the time of the study) were outpatients of the pediatric diabetes clinics of four tertiary care hospitals in Finland and were contacted by a health care professional of the clinic. Exclusion criteria were a native language other than Finnish and a diagnosed neurological or neurocognitive disorder. Sixty-three children with T1DM and their mothers participated in the study and 16 declined to participate, thus yielding a study participation rate of 80%. The sample was assessed from 2006–2008. The mothers and the children were informed about the study, with the mothers providing written informed consent and the children giving verbal assent. Each mother filled out the Blocks’ Child Rearing Practices Report [28,29] in reference to her child with diabetes while her child had a neuropsychological assessment as a part of a larger study. The child’s medical history was obtained from the medical records and confirmed by the mother, who also provided information on the child’s development, mother’s education and family situation. The protocol was approved by the Kymenlaakso Hospital District’s Ethics Committee, in accordance with the Declaration of Helsinki.

The children who comprised the control group in the Jyväskylä Longitudinal Study of Dyslexia [30,31] (*n* = 92) formed the comparison group for this study. They were drawn from families attending maternity clinics and were screened over 4 consecutive years. The comparison group had been followed from birth in order to have a representative sample of typically developing children when searching for the predictors and precursors of dyslexia. They had neither diabetes nor familial risk for dyslexia, and they all had Finnish as their native language. The educational level of the comparison group parents was equivalent to the educational level of the Finnish adult population. Eighty-three mothers of the comparison group completed the Blocks’ Child Rearing Practices Report [28,29] when their child was nine years of age (participation rate 90%). No differences were observed between the diabetes and comparison groups in the parents’ education or the children’s gender and age (Table 1).

### 2.2. Measures

*Diabetes-related measures.* The following data regarding diabetes management among children with T1D was obtained from the medical records: age at diagnosis of T1D; early glycemic control (A1C one year after diagnosis of T1DM); and current glycemic control (the most recent A1C measurement). Information on diabetes management was recorded prospectively into the child’s medical records at visits to the clinic every 3 months on average.

*Parenting style.* Mothers’ parenting styles were assessed with the Blocks’ Child Rearing Practices Report [28] (Finnish version [29]), which includes 19 statements on a 5-point Likert scale (1 = *not like me at all*, 5 = *very much like me*). The mean scores of Affection (10 items), Behavioral Control (5 items), and Psychological Control (4 items) were used in the analyses. The statements measuring affection included items reflecting a positive relationship with the child (e.g., *“I am easygoing and relaxed with my child”*). The items showing that the child’s behavior would have consequences (e.g., *“My child should learn we have rules in our family”*) measured behavioral control. The items reflecting parental sacrifice or guilt and shame induction (e.g., *“I let my child see how disappointed and ashamed I am if she/he misbehaves”*) assessed psychological control. Cronbach’s alpha in the whole sample was 0.86 for Affection, 0.63 for Behavioral Control and 0.72 for Psychological Control. Similar reliabilities have been reported in other studies using the same questionnaire [4,8,29,30].

### 2.3. Data Analysis

Two kinds of analyses were carried out. First, group differences in parenting styles between the mothers of children with T1D and the mothers of healthy children were analyzed with t-tests and further with a Multivariate General Linear Model including group, child’s gender and group–gender interaction as independent variables and mother’s education as a covariant. The analyses for group differences were performed with SPSS 22.0 (IBM, Armonk, NY, USA, 2013).

Second, path analyses were carried out to examine the extent to which early glycemic control (early A1C) is associated with mothers’ parenting styles (i.e., affection, behavioral control, psychological control) and the extent to which parenting styles are associated with current glycemic control (current A1C), after controlling for the level of early glycemic control. Because the preliminary analyses showed gender differences in the associations of study variables, path analyses were carried out separately for boys and girls. The path analyses were performed using the Mplus statistical package (version 7.0; L. K. Muthén & Muthén, 1998–2015).

## 3. Results

### 3.1. Comparison of Parenting Styles Between Mothers with and Without Children with Diabetes

The means and standard deviations of the study variables are presented separately for diabetes and comparison groups in Table 1. The results showed group differences in psychological control (*t* (142) = −2.83, *p* = 0.005), but not in behavioral control (*t* (142) = 0.33, *p* = 0.740) or affection (*t* (111) = 1.63, *p* = 0.105). Group differences in psychological control remained significant (*F* (1,135) = 6.64, *p* = 0.011) after controlling for the child’s gender and mother’s education. The mothers of children with T1D reported higher use of psychological control than the mothers of children without diabetes. Gender X Group interaction was not significant, suggesting that there was a group difference in psychological control independent of the child’s gender.

### 3.2. Relationships Between Mother’s Parenting Styles and Child’s Glycemic Control

Path models were constructed to examine the extent to which early glycemic control would be associated with the mothers’ subsequent parenting style dimensions, and the extent to which parenting style dimensions would be associated with concurrent glycemic control after controlling for the level of early glycemic control and the mothers’ level of education. The analyses were carried out separately for girls and boys. Correlations between predictors and outcome variables of the path models are presented separately for boys and girls in Table 2.

The results for girls showed, first, that the level of early A1C was positively and statistically significantly (*standardized estimate* = 0.27, *p* < 0.05) associated with maternal psychological control: The poorer the girls’ early glycemic control, the more psychological control mothers applied. Early glycemic control was not associated with mothers’ affection or behavioral control. The results showed further that mothers’ behavioral control was positively associated with current A1C after controlling for the level of early glycemic control and maternal education: The higher the level of behavioral control, the poorer the current glycemic control (*standardized estimate* = 0.23, *p* < 0.05).

The results for boys showed, first, that early glycemic control was not associated with mothers’ parenting styles. However, mothers’ psychological control was statistically significantly associated with boys’ current A1C: The higher the level of psychological control mothers deployed, the poorer the boys’ current glycemic control was (*standardized estimate* = 0.31, *p* < 0.05), after controlling for the level of early glycemic control and maternal education.

Because it is possible that the type of insulin treatment impacts on the results, t-tests were carried out to compare children with different types of treatment, i.e. injections (*n* = 52) and insulin pump (*n* = 11), according to glycemic control and parenting style variables. The results revealed, however, that the type of treatment was not related either to glycemic control (*t* (61) = −0.06, *p* = 0.951) or parenting styles (*t* (61) = 1.53, *p* = 0.131 for affection, *t* (61) = 1.11, *p* = 0.271 for behavioral control, *t* (60) = 1.08, *p* = 0.286 for psychological control).

## 4. Discussion

The present study examined the differences in parenting styles between the mothers of children with and without type 1 diabetes, and whether parenting styles are associated with the child’s glycemic control. The results showed that the mothers of children with T1D used more psychological control than the mothers of healthy children. Moreover, mothers’ greater use of psychological control was associated with poorer current glycemic control among boys with T1D. Among girls, however, poor glycemic control at the early stage of disease was associated with mothers’ greater use of psychological control, whereas the greater use of behavioral control was associated with poorer concurrent glycemic control.

This study was the first to show that mothers of school-age children with T1D use more psychological control with their children than mothers of healthy children. Graue and colleagues [17] had a similar observation from the adolescents’ perspective. Our study, thus, reveals that reliance on stronger psychological control in mothers of children with T1D is already present before adolescence, suggesting long-lasting effects on the mother–child relationship. This is understandable, as fear of complications and the burden of continuous care are expected to cause stress and increase the use of control [2,32,33] in parents with a child with T1D. However, psychological control—for example over-protectiveness, criticism, and guilt induction—can be harmful for children and has been shown to be associated with poorer well-being of the child [4,8,10,13,15,34]. Although differences in parenting styles between parents of chronically ill and healthy children may seem obvious, they have not yet been extensively studied. New information about specific problems in parenting may lead to development of new interventions for parents with a chronically ill child.

The results of the study also suggest that parenting styles may have a reciprocal relationship with a child’s glycemic control. First, girls’ poorer glycemic control at the early stage of disease was associated with their mothers’ subsequent greater use of psychological control. This finding is in accordance with the previous research [2] and might be due to the fact that when a mother worries over her child’s health increases, she sees the child as more vulnerable [35] and, therefore, uses overprotection and other maladaptive aspects of control.

Second, among boys, the mother’s greater use of psychological control was associated with poorer concurrent glycemic control, after controlling for early glycemic control. Most previous studies have not been able to show that psychological control is associated directly with glycemic control [15,16,17,24]. However, it has been associated with poorer diabetes self-care behavior [12,24] and well-being [12,13,15,17,24], which, in turn, are related to poorer diabetes care. This study confirmed the result by Goethals et al. [25] that psychological control is associated with poorer glycemic control. However, the possible mediating factors include problems in mood, behavior and adherence to diabetes care. Also, the child’s poorer diabetes care may increase the parent’s distress, which may increase the use of psychological control.

Third, among girls, the greater use of behavioral control was associated with poorer glycemic control, which was an unexpected finding. Most previous studies have found that behavioral monitoring and control is related to better diabetes care [14,16,23]. It has, however, been reported that adolescent girls are sensitive to the use of parental control [16], and it has been suggested that this may influence their diabetes self-care. Our study implicates that a similar tendency of sensitivity to parental control may be present already in school-age girls. Behavioral control that is intended to set rules and monitor the child’s behavior is different from control that intrudes on the child’s psychological autonomy. It is important to study both types of control, because they might have different effects on the child’s well-being, on interactions between parent and child, and eventually on diabetes care, which may suffer from problems of interaction.

Before generalizing these results, though, the following limitations of the present study should be taken into account. First, the reliability of the behavioral control scale was rather low, and therefore the results concerning behavioral control must be treated with caution. For example, the observation regarding negative effects of behavioral control on diabetes care in girls should be replicated in other studies using reliable questionnaires for the different types of parental control. Second, the study was not able to use children’s appraisals of parenting styles. However, it is also important to identify the parents’ opinions of their parenting. Interestingly, the mothers in this study reported using maladaptive parenting (e.g., psychological control), although previous studies have not been able to show signs of maladaptive parenting in parent reports [18,19]. When considering parenting styles as dimensions (e.g., affection, psychological and behavioral control) rather than typologies (e.g., authoritarian, authoritative) differences in the use of parenting styles and their role in diabetes care could be found. Third, the sample in this study was small, and thus its statistical power was not sufficient to test multiple interactions. For example, individual features and the well-being of the mother and child, family structure and resources have an effect on parenting and diabetes care, but this study could not include these factors in the analyses. Consequently, there is a need to replicate the study with a larger sample. Finally, parenting styles were measured only once, and thus no causal conclusions about the direction of effects can be drawn. Further studies using a cross-lagged longitudinal design assessing both glycemic control and parenting styles at several measurement points are needed to examine the direction of effects and reciprocal interactions between parenting styles and children’s health and development.

Earlier research on parenting children with T1D has concentrated mainly on adolescents. Because there are different demands for maturity and independence at different developmental stages, it is important to study the phenomena also among younger children. The present study adds to previous literature by showing that also the mothers of younger children with T1DM show higher levels of psychological control than the mothers with healthy children and, furthermore, that this heightened parental psychological control is associated with poorer outcomes in diabetes care. These results are quite similar to those reported with adolescents [16]. Although the conflicts over parental control and child’s autonomy in diabetes self-care often escalate during adolescence, excessive parental control in younger children may as well be harmful to diabetes care and reflect interaction problems in the family. An education program for parents about child development, behavior management, and transition of diabetes-care responsibilities would perhaps diminish conflicts over diabetes during adolescence [36]. There already are promising interventions for parenting and, for example, Triple P-Positive Parenting Program has also been studied in families with diabetes [37,38]. However, further research is still needed for developing interventions for parenting chronically ill children.

## 5. Conclusions

The results of the present study suggest that psychological control in families with diabetes needs attention, because it was shown to be associated with poorer diabetes care. Clinicians should pay attention to the quality of interaction between the child and the parents and notice the use of excessive control (for example overprotection, shame and criticism) as a sign of maladaptive parenting, which may influence the child’s and the parent’s well-being and diabetes care.

## Figures and Tables

**Table 1:** Participant characteristics in the diabetes and comparison groups.

Variable	Diabetes Group 31 Girls, 32 Boys		Comparison Group 36 Girls, 47 Boys	
	Mean (*SD*)	Range	Mean (*SD*)	Range
Child’s age (year)	9.9 (0.3)	9.3–10.6	9.8 (0.3)	9.2–10.4
Mother’s education ^a^	4.3 (1.3)	2–7	4.6 (1.4)	2–7
Parenting Style				
Affection	4.3 (0.5)	2.9–5.0	4.4 (0.4)	3.3–5.0
Behavioral control	3.8 (0.5)	2.3–4.8	3.8 (0.5)	2.4–5.0
Psychological control	2.8 (0.6)	1.3–4.5	2.5 (0.6)	1.0–3.8
Age at diagnosis (year)	2.9 (1.3)	0.6–5.0	-	
Early A1C (mmol/mol)	64 (10)	46–91	-	
Current A1C (mmol/mol)	67 (8)	45–90	-	

Note. Early A1C, A1C 1 year after diagnosis; Current A1C, the most recent A1C value. ^a^ Mother’s education was classified using a 7-point scale constructed by combining general education, secondary vocational education, and tertiary education.

**Table 2:** Correlations between parenting style and diabetes-related variables for girls (above the diagonal) and boys (below the diagonal) with T1D.

Variable	1.	2.	3.	4.	5.	6.
1. Affection	-	−0.05	−0.41 *	0.21	0.01	0.40 *
2. Behavioral control	−0.02	-	0.32	0.18	0.23	0.02
3. Psychological control	0.05	0.45 *	-	0.25	−0.05	−0.26
4. Early A1C	0.16	0.09	0.09	-	0.20	0.07
5. Current A1C	0.02	0.03	0.23	0.50*	-	0.25
6. Mother’s education	0.20	0.01	0.07	-0.12	−0.39*	-

Note. Early A1C, A1C 1 year after diagnosis; Current A1C, the most recent A1C value. * *p* < 0.05.
